# Following “the Roots” of Kratom (*Mitragyna speciosa*): The Evolution of an Enhancer from a Traditional Use to Increase Work and Productivity in Southeast Asia to a Recreational Psychoactive Drug in Western Countries

**DOI:** 10.1155/2015/968786

**Published:** 2015-11-10

**Authors:** Eduardo Cinosi, Giovanni Martinotti, Pierluigi Simonato, Darshan Singh, Zsolt Demetrovics, Andres Roman-Urrestarazu, Francesco Saverio Bersani, Balasingam Vicknasingam, Giulia Piazzon, Jih-Heng Li, Wen-Jing Yu, Máté Kapitány-Fövény, Judit Farkas, Massimo Di Giannantonio, Ornella Corazza

**Affiliations:** ^1^Centre for Clinical & Health Research Services, School of Life and Medical Sciences, University of Hertfordshire, College Lane Campus, Hatfield, Herts AL10 9AB, UK; ^2^Department of Neuroscience, Imaging and Clinical Sciences, Gabriele D'Annunzio University, Chieti, Italy; ^3^Centre for Drug Research, Universiti Sains, Penang, Malaysia; ^4^Institute of Psychology, Eötvös Loránd University, Budapest, Hungary; ^5^London School of Economics and Political Science, LSE Health and Social Care, London, UK; ^6^Department of Psychiatry, University of Cambridge, Cambridge, UK; ^7^Department of Neurology and Psychiatry, Sapienza University of Rome, Rome, Italy; ^8^School of Pharmacy and Ph.D. Program in Toxicology, Kaohsiung Medical University, Kaohsiung, Taiwan; ^9^Faculty of Health Sciences, Semmelweis University, Budapest, Hungary; ^10^Nyírő Gyula Hospital, National Institute of Psychiatry and Addictions, Budapest, Hungary

## Abstract

The use of substances to enhance human abilities is a constant and cross-cultural feature in the evolution of humanity. Although much has changed over time, the availability on the Internet, often supported by misleading marketing strategies, has made their use even more likely and risky. This paper will explore the case of *Mitragyna speciosa* Korth. (kratom), a tropical tree used traditionally to combat fatigue and improve work productivity among farm populations in Southeast Asia, which has recently become popular as novel psychoactive substance in Western countries. Specifically, it (i) reviews the state of the art on kratom pharmacology and identification; (ii) provides a comprehensive overview of kratom use cross-culturally; (iii) explores the subjective experiences of users; (iv) identifies potential risks and side-effects related to its consumption. Finally, it concludes that the use of kratom is not negligible, especially for self-medication, and more clinical, pharmacological, and socioanthropological studies as well as a better international collaboration are needed to tackle this marginally explored phenomenon.

## 1. Introduction

Kratom (*Mitragyna speciosa* Korth., of the Rubiaceae family) is a 4–16-metre high tropical tree, indigenous to Southeast Asia, the Philippines, and New Guinea. Traditionally, in certain regions of Southeast Asia, the chopped fresh or dried leaves of the tree are chewed or made into tea by local manual labourers to combat fatigue and improve work productivity [1]. In addition, kratom preparations have also been used for centuries during socioreligious ceremonies and to treat various medical conditions, such as morphine dependence in Thailand [2], and as opium substitute in Malaya [2]. It has been suggested that the genus was given the “*Mitragyna*” name by the Dutch botanist Korthals because the leaves and the stigmas of the flowers of the plant resemble the shape of a bishop's mitre [3]. However, considering its variety of uses, it could be speculated that the term derives from the “Mithraic cults,” seen as a source of spiritual transcendence for thousands of years [4].

**Figure 1:**
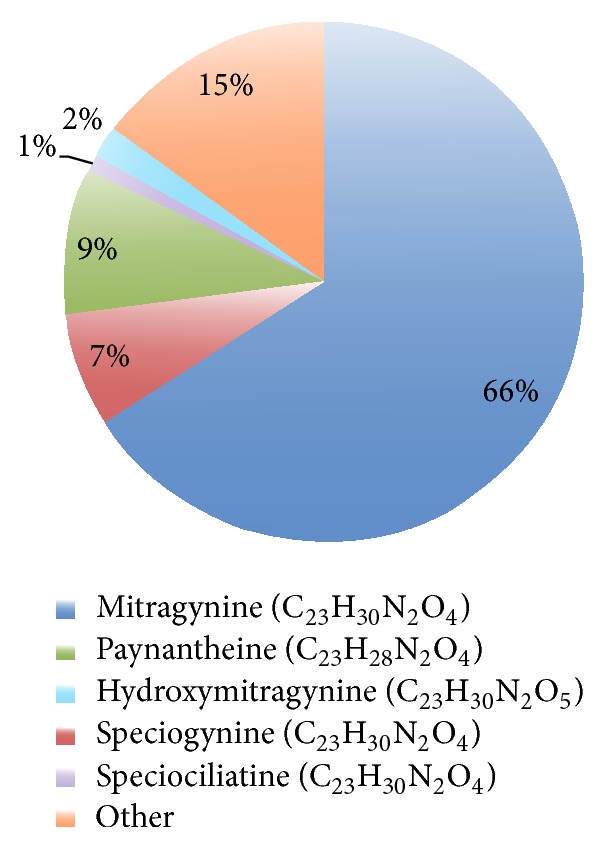
An estimate of Thai kratom extract composition. The phytochemicals isolated from various parts of the tree include overall 40 structurally related alkaloids as well as several flavonoids, terpenoid saponins, polyphenols, and various glycosides.

Kratom preparations contain varying amounts of several phytochemicals, making their pharmacological and toxicological evaluation unique and difficult (Figure 1). The main psychoactive components in the leaves are alkaloids mitragynine and 7-hydroxymitragynine both found only in* Mitragyna speciosa, *but other analogues have been identified (e.g., speciogynine, paynantheine, and speciociliatine) [3, 4] (Figure 1). The effects of kratom in humans are dose-dependent where small doses produce stimulatory effects resembling the stimulant effect of drugs such as cocaine or amphetamines, while larger dosages tend to be associated with sedative-narcotic effects that resemble drugs such as opiates [5].

Imported to Western countries from Southeast Asia, kratom has become in recent years a popular enhancer, which could also be classified as novel psychoactive substance (NPS) [3]. Beyond kratom, some of the most widely used psychoactive plants, largely not under international control, include* Salvia divinorum*, khat (*Catha edulis*), Hawaiian baby woodrose seeds (*Argyreia nervosa*), fly agaric (*Amanita muscaria*), “Magic Mushrooms” (*Psilocybe* and related species), Peyote (*Lophophora williamsii*), Ayahuasca (*Banisteriopsis caapi* and* Psychotria viridis*), and “Genie” (a smoking mixture containing multiple plant materials and of dubious pharmacognostical identity) [6]. Natural products often used as enhancers are exceptionally complex in terms of their chemistry. This adds complexity of their pharmacological effects, with a paucity of data relating to the toxicology of these materials, and even less regarding their interactions with conventional drugs of abuse [7]. This is further complicated by the possibility of adulteration processes [8]. The level of complexity, variability, and the unknown nature of these samples, coupled with the risks associated with taking psychoactive materials, could offer further risks of ill health by misadventure, with potentially life-threatening consequences [9]. In this scenario, predictions of novel psychoactive drug trends in Western countries specifically suggest that kratom use will increase in the next years [10].

The aim of the present study was to study this new phenomenon by (i) reviewing the state of the art on kratom pharmacology and identification; (ii) providing a comprehensive overview of kratom use cross-culturally, ranging from its traditional use in native societies in Southeast Asia to its more recent diffusion as a NPS in Western countries; (iii) exploring the subjective experience of users; (iv) identifying risks and side-effects related to its consumption.

## 2. Materials and Methods

A collaborative and multidisciplinary effort to study the rapid diffusion of kratom was carried internationally by ten research centres: the School of Life and Medical Sciences, University of Hertfordshire (United Kingdom), Department of Neuroscience, Imaging and Clinical Sciences, Gabriele D'Annunzio University, Chieti (Italy), Centre for Drug Research, Universiti Sains, Penang (Malaysia), Institute of Psychology, Eötvös Loránd University, Budapest (Hungary), London School of Economics and Political Science, LSE Health and Social Care, London (United Kingdom), Department of Psychiatry, University of Cambridge, Cambridge (United Kingdom), Department of Neurology and Psychiatry, Sapienza University of Rome, Rome (Italy), School of Pharmacy and Ph.D. Program in Toxicology, Kaohsiung Medical University, Kaohsiung (Taiwan), Faculty of Health Sciences, Semmelweis University, Budapest (Hungary), and Nyírő Gyula Hospital National Institute of Psychiatry and Addictions, Budapest (Hungary). A review of the literature (1967–2015) on kratom and its main components was performed in three databases: PsycINFO, PubMed, and Medscape. Keywords used to carry out the database searches included the following: “kratom”, “*Mitragyna speciosa”, *“mitragynine”, and “7-hydroxymitragynine”. Peer-review data that emerged from the search were integrated with an exploratory qualitative assessment of 227 websites, drug fora, and other online resources (i.e., e-newsgroups, chat-rooms, mailing lists, e-newsletters, and bulletin boards). This was carried out on a regular weekly basis (between January 2008 and April 2015) using the Google search engine in three languages (English, Italian, and Hungarian). Once the substance availability of information was identified, further specific searches were carried out for narratives focusing on the following issues: (i) motivations behind its recreational use and possible trends of misuse; (ii) the nature of its effects on users, including adverse reactions and polydrug misuse/idiosyncratic combinations; (iii) any other relevant information. For the purpose of reporting the results in this paper, any data collected from online fora, such as usernames and complete URLs for specific threads that were considered personal identifiable, were anonymized. Additional searches were conducted using the Global Public Health Intelligence Network (GPHIN), a secure Internet-based early warning system developed by Health Canada and the World Health Organization (WHO), which monitors media reports in six languages, Arabic, Chinese, English, French, Russian, and Spanish.

Permission for the study was granted by the School of Pharmacy Ethics Committee, University of Hertfordshire, Hatfield, United Kingdom (November 2013; PHAEC/10-42).

## 3. Results

113 studies emerged from the literature review and were critically analysed. Among these, 18 results were considered not relevant (resulting duplicated, botanical studies, or studies focusing mainly on other selected chemical compounds) and therefore excluded. The remaining 95 articles were further qualitatively analysed and thematically divided in three main areas of interest related to* Mitragyna speciosa* and its main constituents: (1)* in vitro* and preclinical data on pharmacology and behavioral effects (*N* = 51), (2) laboratoristic techniques for identification/characterization (*N* = 26), and (3) epidemiological/toxicological reports on humans (*N* = 18). Data on kratom that emerged from the online searches were identified, monitored, and registered into 5 categories: (1) epidemiology and motivation of use; (2) legal status, methods of purchase, and typical price; (3) forms of kratom use; (4) subjective pleasurable effects, adverse effects, and fatal intoxications related to kratom; (5) pattern of polyabuse. The results from the review of scientific literature and online sources were comprehensively integrated and summarized in the following three main subsections: (i) preclinical data about pharmacology and identification of kratom constituents; (ii) kratom use in humans in Southeast Asia; (iii) kratom use in humans in Western countries.

### 3.1. Pharmacology and Identification of Kratom Constituents

Kratom has both opioid- and psychostimulant-like subjective effects [11]. The phytochemicals isolated from various parts of the kratom tree include over 40 structurally related alkaloids [7] of which mitragynine (Figure 1) is the most important with up to 66% purity in the extract of leaves from Thailand, and only 12% in kratom leaves from Malaysia. This alkaloid is the one responsible for analgesic activity that has been linked to kratom use mostly due to its potent opioid agonist property [12]. Although mitragynine can act on the mu (*μ*), kappa (*κ*), and delta (*δ*) opioid receptors, it is structurally different from morphine and other components from the opioid family; the reason why it has been suggested is that it might also present with a more broad receptor binding activity [13]. Mitragynine and its analogues in kratom (including speciogynine (7%), paynantheine (9%), and speciociliatine (1%) (Figure 1)) are indole alkaloids of the* Corynanthe*-type, possessing a monoterpene (iridoid) moiety [12]. Differently, 7-hydroxymitragynine (Figure 1), a minor constituent (2%) of* M. speciosa*, when isolated demonstrates a potent antinociceptive activity in mice [14]. It is now considered to be a major contributory factor for the analgesic properties of* M. speciosa* due to its selectivity for *μ*- and *κ*-opioid receptors [7]. The presence of a hydroxyl group at C-7 increases the potency of 7-hydroxymitragynine to be 13- and 46-fold higher than morphine and mitragynine, respectively, both* in vitro* and* in vivo* [12, 15, 16]. This might be one of the main pharmacological markers of kratom products' quality and potency. Recent studies further revealed how complex is kratom's pharmacology, involving a *κ*-opioid and dopamine D1 receptors interaction in its various effects [17]. Serotonergic and adrenergic pathways have also been involved in the effects of mitragynine, mostly due to its broad affinity to different receptors [18]. Indeed, the pharmacological mechanisms responsible for stimulant activity are yet to be clearly established [19]. Another confounding factor might be the action of some other isolated compounds (speciociliatine, speciogynine, and paynantheine), for example, whose effects were not inhibited by naloxone in animal studies [12, 15].

It is possible that mitragynine is relatively safe at lower subchronic dose but exhibits toxicity at a highest dose [20]. However, the erratic pharmacology of kratom makes it difficult to define a specific dose threshold. According to online reports and traditional experiences, subjective effects of kratom depend on the dosage: at low to moderate dose (1–5 g) it has a mild pleasant stimulant effect; at moderate-high dose (5–15 g) the compound has opioid-like analgesia and sedation [20, 21]. No studies have been conducted so far to determine the blood concentration in patients, and future approach should consider this point in order to prevent overdose, considering the possible risk of no response to naloxone [22, 23]. The standard half-life of mitragynine is 3.85 ± ~1 hr, depending upon the individuals natural levels of enzymes and other factors [24, 25]. 7-Hydroxymitragynine has quite a bit shorter duration, with an average half-life of 2.5 ± 0.7 hours [24, 25]. Recent evidences suggest that the hydrophobicity, poor water solubility, high variability of drug release in simulated biological fluids, and acid degradable characteristics of mitragynine probably further influence the large variability of its pharmacological responses reported in the literature [24]. This was confirmed by liver, kidney, and brain histopathological changes, as well as hematological and biochemical changes in mice [20]. Mitragynine, 7-hydroxymitragynine, and mitraphylline exhibit high plasma protein binding (>90* *%) determined by equilibrium dialysis [25]. The metabolism of kratom is mainly hepatic. While it seems that kratom is unlikely to have any significant clinical effects on CYP3A4 activity, on the other hand mitragynine might inhibit other cytochrome P450 enzyme activities, specifically CYP2D6 [26, 27]. These data indicate the possibility of a drug interaction if mitragynine and 7-hydroxymitragynine are coadministered with drugs that are P-glycoprotein substrates [27].

Current preclinical information on kratom suggests that this ethnodrug, containing several different active alkaloids, has a harmful toxicological profile and must be studied in detail in order to better define its potential as therapeutic drug [28]. Acute administration of mitragynine produces anxiolytic-like effects attributed to the interactions among opioidergic receptor systems [29]. Other authors attempted to reveal a possible link with the stress-related corticotropin pathway [30]. Other evidences show that mitragynine exerts an antidepressant effect in animal behavioral model interacting with neuroendocrine HPA axis systems [31]. Among its potential benefits, in addition to analgesic activity [13, 32], mitragynine seems to be also a key component for the anti-inflammatory properties of kratom by suppressing prostaglandin E2 (PGE-2) production in the cyclooxygenase 2 (COX-2) pathway [16]. Moreover, some authors claim that kratom might be promising antioxidant and anticancer or chemopreventive compounds [33]. Kratom extracts and mitragynine have been shown to possess cytotoxicity to some human cancer cell lines, namely, SH-SY5Y cells (neuronal cells), [34] and avoid the tolerance and dependence on chronic morphine treatment in mice as well as in human neuroblastoma SK-N-SH cell [35, 36]. Other interesting properties of the compound might be the capability to modulate muscle neurogenic contraction [37–39] and gastric secretion [40].

While kratom metabolites could have the potential to be developed as new therapeutic agents, there are also possible serious adverse effects of these materials under investigation. There have been different studies showing serious conditions after repeated administration as elevated blood pressure, nephrotoxic effects [41], impaired cognition and behaviour [42, 43], dependence potential [42], and hepatic failure [41, 44]. The onset of liver injury is described to occur within 2 to 8 weeks of starting regular use of kratom powder or tablets, with symptoms of fatigue, nausea, pruritus, and dark urine followed by jaundice [41, 44]. The pattern of liver injury seems to be typically cholestatic and can be severe with serum bilirubin levels rising above 20 mg/dL [44]. Kratom constituents were also identified to be potentially cardiotoxic, ideally potentiating Torsade de Pointes through inhibition of rapid delayed rectifier potassium current (IKr) in human cardiomyocytes [45].

At present, kratom constituents are not detected by conventional drug screening tests: advanced tests like liquid chromatography-tandem or ion-mass spectrometry are required [7].

### 3.2. Kratom Use in Southeast Asia 

#### 3.2.1. Diffusion and Modalities of Consumption

*Mitragyna speciosa* (Rubiaceae) is an indigenous plant of Southeast Asia. This herbal plant is also known as “kratom,” as “ketum” or “biak” (Malaysia), or as “krathom” (Thailand, “thom” in Southern Thailand) and has been used for millennia (a) as a stimulant; (b) as a remedy in traditional medicine; and (c) in social context [46, 47]. Historically, manual labourers (e.g., fisherman, farmers, and rubber-tappers) in northern Malaysia and southern Thailand commonly used ketum leaves to improve their work productivity under the sweltering sun and to relieve fatigue [1]. Rural folk have traditionally ingested ketum leaves to self-treat common medical problems (e.g., diabetes, diarrhoea, fever, and pain) and used it as a wound poultice [46, 48]. Ketum was also used as an opium substitute in Malaya during opium scarcity [47]. It is still popularly consumed in Asian communities during social gatherings in the village [47].

Traditionally, the fresh or dried leaves of kratom are chewed or brewed into tea or smoked [49]. Ketum is bitter and sugar or sweet beverages are commonly added to mask its taste [49]. To experience vigour and euphoria, traditional “kratom eaters” chew one to three fresh leaves at a time [1, 2]. Regular and addicted users chew 3–10 times a day [1]. Amattayakul [50] reported that an average green leaf weighs about 1.7 g and a dry leaf about 0.43 g and twenty kratom leaves contain about 17 mg of mitragynine; no information is available on other active compounds of the plant. Ketum is currently widely available in many Asian countries (e.g., in Malaysia) where it can be easily bought from ketum traders in the community [1]. Consumers can be classified into two main groups: the first includes those who solely use ketum to improve physical tolerance to laborious work and the second polydrug users who attempt to manage drug withdrawal symptoms or reduce the intake of other opiates like heroin [51]. A recent study showed that out-of-treatment opiate users in Malaysia often use ketum to reduce their dependence on illicit opiate as well as to ameliorate opiate withdrawal symptoms [51].

At present, there is no systematic data on the prevalence of ketum use in all the native countries, but it seems to be considerable in Malaysia and Thailand. A survey performed in 2007 investigating kratom use in Thailand (26,633 respondents aged 12–65 years) indicated that the lifetime, past year, and past 30 days prevalences for kratom were 2.32%, 0.81%, and 0.57%, respectively [3]. These figures, with the exception of lifetime use, were significantly higher than those for cannabis making kratom the most widely used illicit drug in Thailand. Again in Thailand past 30-day prevalence studies among 13–16-year-old students (*n* = 8 708–12 148) in 2002, 2003, and 2004 showed an increase in the lifetime use of kratom (from 3.97% to 9.43%) [3]. Another study in Thailand in 2006 showed a prevalence of psychoactive drugs in 1,635 motor vehicle drivers: 0.9% were positive to mitragynine [52]. Overall, reported seizures linked to kratom quintupled in Thailand from 2005 to 2011, far higher than those reported for any other drug [6]. Kratom reported seizures in Malaysia and Myanmar also reached record levels in 2011, at roughly one ton each [6]. Conversely, in Taiwan, heroin, methamphetamine, and some new drugs such as synthetic cathinones (methylone, mephedrone, and MDPV) and synthetic cannabinoids (K2) have been identified since 2000s according to the* Substance Abuse Monitoring and Reporting Systems *(SAMRS), which is a national data collection system for substance abuse [53]. However, use of kratom and its major alkaloid mitragynine has not been detected via the collected information from the SAMRS [53]. Therefore, it could be suggested that kratom may not yet be a drug of choice in Taiwan and might still be considered a rather culture-bounded phenomenon in Asian countries.

The possession of kratom leaves has been illegal in Thailand since 1943 [49]. Kratom is also controlled in a few other countries in the region (Malaysia and Myanmar) and elsewhere (Australia, Bhutan) [6]. In parallel, kratom-related arrests more than doubled between 2007 and 2011 in both Myanmar and Thailand [6]. To control its widespread abuse, ketum was banned in Malaysia and regulated under the Poisons Act 1952 [49]. Those caught for possessing or processing ketum leaves can be fined for approximately RM 2,000 (US 450) or imprisoned if found guilty [49]. The Malaysian government is in the midst of regulating ketum under the Dangerous Drugs Act 1952, which will consider the substances as harmful as opiates and amphetamines [49].

#### 3.2.2. Stigma and Side-Effects

**Figure 2:**
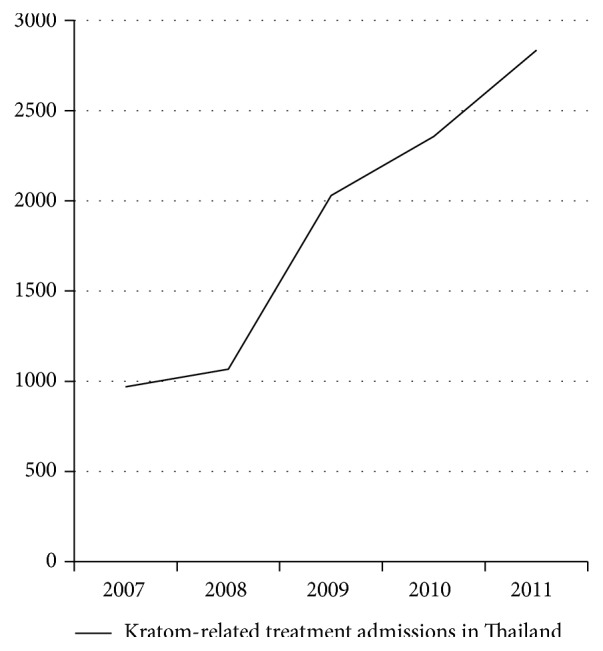
Kratom-related treatment admissions in Thailand almost tripled between 2007 and 2011. Source: United Nations Office on Drugs and Crime, Patterns and Trends of Amphetamine-Type Stimulants and Other Drugs: Asia and the Pacific—2012 (Bangkok, 2012).

Among rural folk, the believe that kratom is a better alternative to illicit drugs, such as heroin and methamphetamine, is still diffuse and it is mainly used for its invigorating-like effects [54, 55]. Five to ten minutes after kratom consumption users describe themselves as feeling happy, strong, and active, especially among those working in the agricultural sector [1]. They claim that “their mind is calm” after the consumption of the drug [1]. Overall, there is no real social stigma towards ketum users and being dependent on ketum is not seen as a major problem or taboo in Malaysia, at least for men. Apparently, society accepts male addicts who work to support their family but do not accept female addicts [1]. Moreover, it seems that ketum dependents are not neglecting their family and the impairment of their social functioning is still under debate [56]. A recent cross-sectional survey in three northern states of Peninsular Malaysia investigated 293 regular kratom consumers [54]. Findings showed that regular kratom users do not seem to experience major impairments in their social functioning, despite being dependent on kratom for prolonged periods [54]. Furthermore, ketum use does not imply risky behaviours such as needle sharing, common in heroin dependents [57]. On the other hand, evidence shows that kratom can generate addiction problems and lead to other social issues [54]. Considering how kratom use is consistent, figures for treatment admissions for its use appear rather low, accounting for, for example, 2 percent of all drug treatment admissions in Thailand in 2011 (Figure 2). However, they are declared to be considerably on the rise [6]. Kratom-related treatment admissions almost tripled between 2007 and 2011 (Figure 2) [6]. This could be also partially due to a more strict antidrug policy, where individuals caught with kratom are obliged to engage in treatment programs. Surely, findings show that regular kratom use is associated with drug dependency, development of withdrawal symptoms, and craving [54]. Many regular users declare their difficulty to abstain from kratom use and experiencing sharp unpleasant symptoms during abstinence periods [58]. Physical withdrawal symptoms include anorexia, weight loss, decreased sexual drive, insomnia, muscle spasms and pain, aching in the muscles and bones, jerky movement of the limbs, watery eyes/nose, hot flushes, fever, decreased appetite, and diarrhoea [48, 54]. Psychological withdrawal symptoms commonly reported are nervousness, restlessness, tension, anger, hostility, aggression, and sadness [1, 54]. Long-term addicts are described to become thin and have skin pigmentation on their cheeks, due to the capacity of mitragynine to increase the production of melanocytes-stimulating substance [1, 46]. Regular ketum use is also reported to cause psychotic symptoms such as mental confusion, delusion, and hallucination [1].

Regarding polydrug abuse, in Asia, patterns of complex cointaking involving kratom are also reported. Beyond “classic” substances and many NPS as synthetic phenylethylamines and cathinones, peculiarly there have been cases where codeine is added into kratom drinks to obtain a better “high” or euphoria. In southern Thailand, in recent years, homemade ice-cold cocktails, called “4 × 100,” have become popular for their alleged alcohol-mimicking effect among young Muslim people [3]. The cocktails are made from kratom leaves, a caffeine-containing soft drink, and codeine- or diphenhydramine-containing cough syrup as the three basic ingredients to which ice cubes, an anxiolytic, an antidepressant, or an analgesic drug is added [3, 59].

So far, there have not been any mortality or toxicity incidents directly related to ketum use reported in Asian countries. One possibility is that ketum users in Asia normally buy fresh ketum juice, from a local known supplier in the rural area, unlikely to be an adulterated preparation. Traditionally, ketum in Asia has been used for its stimulant effects and the dose consumed might be lower than the one consumed for recreational purposes [19]. But it is also possible that local health care providers in Asia, perceiving ketum as a safe traditional herbal drink, might not attribute some of the medical problems reported by users to ketum use; this factor may indeed contribute to the underreporting of adverse effects among ketum users in Southeast Asia.

### 3.3. Kratom Use in Western Countries

#### 3.3.1. Diffusion, Modalities, and Reasons of Consumption

In recent years kratom has become popular in the EU, US, and other countries (e.g., Japan) as a recreational novel compound [3, 6, 60]. A variety of* Mitragyna speciosa* related products are easily accessible from local smart shops and increasingly available for sale on the Internet, in particular on web based “legal highs” pharmacies, but their exact content is not always verified [61, 62]. Many different formulations are available, including raw leaves, capsules, tablets, powder, and concentrated extracts [49]. Prices vary between countries, depending on the type and amount of the purchased product, for example, ranging from 2 to 10 euros per gram for “kratom 15X” extracts, 6 to 15 euros per 10 gram for dried kratom [49], or sometimes even for lower prices (from less than 1 euro per gram for “kratom power”) [21, 63–65].

*Mitragyna speciosa* and/or mitragynine and/or 7-hydroxymitragynine are currently controlled only in a small number of EU Member States, such as Denmark, Latvia, Lithuania, Poland, Romania, and Sweden [3]. Kratom is also largely uncontrolled in the US at a federal level while at the state level there are some exceptions such as Indiana, Iowa, Louisiana, and Massachusetts. This means all parts of the plant and its extracts are legal to cultivate, buy, possess, and distribute without a license or prescription, and, when sold as a supplement, sales must conform to US supplement laws [66]. Recently, in February 2014, the Food and Drug Administration (FDA) issued “Import Alert 54-15” that seems to provide customs and border agents broad authority to seize kratom products from a number of suppliers outside the US [66].

As kratom is often not monitored in national drug abuse surveys, there is still little information on prevalence of its use. An initial warning about this phenomenon has been launched by the Drug Enforcement Administration (DEA) as early as 2005 [67]. Internet surveys conducted by the EMCDDA in 2008 indicated that kratom was one of the most widely offered “legal highs” in 44% of the investigated 27 online shops across the EU [3]. A more extensive EMCDDA Internet survey in July 2011 showed that kratom was the most widely offered product with 128 out of 631 (or 20%) of online retailers shipping it to the EU. A further online study identified 314 online shops selling NPS that would dispatch products to at least one EU Member State (United Kingdom appeared to be the most common) [49]. Kratom and* Salvia divinorum* were the most frequently offered NPS, available in 92 and 72 online shops, respectively [3]. In 2012 the term “kratom” was found in more than two million results. Of the first 100 websites listed in the search results, 78 were primarily focused on the sale of kratom, while 22 were focused on disseminating information about kratom through the use of discussion boards [5].

#### 3.3.2. Subjective Experience: Online Reports

Since 2001, an exponential growing number of kratom's subjective experiences have been posted online by users (Box 1) [64, 68–72]. Kratom can be smoked, but according to users this has no advantage over chewing or making a tea: the amount of leaves that constitutes a typical dose is too much to be smoked easily [71, 72]. A paste-like extract can be prepared by lengthy boiling of fresh or dried leaves and the syrup produced can be mixed with finely chopped leaves of the palm, made into pills and smoked in pipes (“madatin”) [26]. Small pellets of this extract can be ingested, or again the compound can be dissolved in hot water and consumed alone or mixed with other ordinary herbal teas to make it more palatable (the so-called toss and wash) [27, 64, 69]. Other users prefer alcoholic beverages or to ingest it with food, mixing it with yoghurt or preparing cookies, in order to contrast the bitterness of the compound [71, 72]. Regarding desired/recreational effects of kratom, users report that at low doses it is rather stimulant, mind is “more alert,” physical energy and sometimes sexual arousal are increased, and ability to do physical work may be improved and they also described “entactogenic” effects, like empathy and euphoria (Box 1) [68, 70, 71]. Some people find this level edgy rather than pleasant [68, 69]. At higher doses, experiences describe it as more sedative and analgesic; users prefer to be less sensitive to physical or emotional pain, to feel and look calm, and to have a general feeling of comfortable pleasure [68, 70]. Others report an increase of empathy feelings (Box 1) [68].

#### 3.3.3. Side-Effects

A variety of less explored side-effects experienced by users also emerged from our work (Table 1). These frequently include nausea, constipation, sleep problems, temporary erectile dysfunction, itching, and sweating and also hyperpigmentation and tremor and anorexia and weight loss in long term [64, 68–72]. Some users describe hair loss, probably related to a regular (daily) use of kratom [69]. Withdrawal symptoms are also common, including muscle aches, irritability, mood disturbances, runny nose, diarrhoea, and muscle jerking (Table 1) [70]. Users describe tolerance (requiring the consumption of higher doses to achieve the same effects) and also a “cross-tolerance” to both kratom and opiates after repeated intake [64, 68–72]. Moreover, kratom is increasingly purchased from Internet sources for self-medication [3, 61], especially by individuals with chronic pain to self-manage opioid withdrawal [61, 69] or heroin, methadone, or suboxone withdrawal symptoms [68], or for its anxiolytic and antidepressant effects [73]. For this reason, it is often advertised online as a cheaper alternative to traditional opioid replacement therapies with no need of medical prescription [28]. This could pose a serious problem for doctors prescribing pain medication or opioid substitution therapy to someone who is a regular kratom user. Adverse effects and intoxications cases across various countries have also been reported, including liver toxicity, seizure, and coma [60, 74–76], reports of patients suffering from intrahepatic cholestasis after two weeks of kratom use [44], Adult Respiratory Distress Syndrome [77], and hypothyroidism [78] (Table 1). Evidence also suggests that kratom might be a deadly substance when mixed with other compounds (Table 1). Fatalities resulting from the use of a kratom-based product known as “Krypton” have also been reported [79] with 9 documented cases in Sweden [22]. Subsequent forensic studies revealed that Krypton contained high amounts of the exogenous pharmaceutical agent O-desmethyltramadol, an opioid analgesic and the main active metabolite of tramadol, and it had been added to the plant material. The presence of this contaminant in some online products is well documented [8, 22, 79]. Even though mitragynine was also detected in the products, it was not determined how the two substances may have interacted to cause death. Other “deadly cases” are available: an article described a fatal reaction that appeared to be associated with mixing with propylhexedrine (an *α*-agonist and amphetamine-like stimulant, used as decongestant inhalers); another case indicated that a mix of kratom, over-the-counter cold medications, and benzodiazepines was responsible for the death of a 17-year-old boy; a postmortem detection of kratom together with venlafaxine, diphenhydramine, and mirtazapine was screened in a 24-year man found unresponsive in bed; a middle aged man in therapy with zopiclone, citalopram, and lamotrigine was found dead at home and postmortem analysis of peripheral blood revealed high concentrations of mitragynine and 7-hydroxymitragynine and therapeutic values of intake of the other compounds [23, 80, 81].

It must also be noted that kratom is commonly taken in combination with a variety of other recreational “classic drugs” (e.g., alcohol, cannabis, benzodiazepines, methadone, cocaine, amphetamine, and hallucinogenic mushrooms) and NPS (e.g., kava, mephedrone, and other synthetic cathinones, tryptamines, and phenylethylamines such as 2C-E, AL-LAD, and 4-HO-MiPT) [68].

## 4. Discussion and Conclusions

Indeed, the use of substances to enhance human abilities is a constant and cross-cultural feature in the evolution of humanity. Opium, coca leaves, mescaline, and various other natural substances have been used for millennia in various cultures for therapeutic purposes, religious ceremonies, and improvement or modification of the physical and mental abilities. Although much has changed over time, the drive for human enhancement has not diminished and drugs availability on the Internet, often supported by misleading marketing strategies, has made their use even more likely and risky [9]. In this context,the tropical tree* Mitragyna speciosa* Korth. (kratom) has now planted its “roots” of use worldwide. Although the phenomenon has only been marginally studied, an exponential number of kratom's subjective experiences have been posted online on drug fora by users in the EU and US and elsewhere. Kratom, still easily available in native countries, is now just “a click” away and potentially available to wide range of new users, including vulnerable individuals. As it emerged from our previous studies [9, 82–88], the web serves also as a repository of information for selected groups, who can share experiences and suggest new products or novel modalities of intake via online fora, chat-rooms, blogs, videos, and others.

Anthropologically, drug addiction history is the complex history of human vicissitude and desire, as human being is a desiring being, trying in every way to assuage sufferings, to enhance feelings of pleasure, and to satisfy inexhaustible and incessant desires [89]. Even today, the line between socially acceptable and unlawful use of a variety of psychoactive products seems to be culture-bound. Kratom is a plant with a well-established traditional use in South Asia to enhance work abilities as well as support traditional medicine and culture, even if officially banned. At the same time, its rapid diffusion in Western societies, where it is often considered a “natural” and thus safer option than illicit drugs or an alternative to opioid treatment, is not devoid of risks. According to preclinical data and case reports published in scientific literature as well as anecdotal experiences posted online, kratom is not a safe drug. Its consumption is associated* per se* with drug dependency, development of withdrawal symptoms, craving, serious adverse effects, and life-threating effects, especially in a multidrug-intoxicating scenario [23, 80, 81, 90]. Furthermore, the idea that legality can equate with the safety of a product might still remain a common insidious misbelief amongst drug users [91]. On the other hand, Suwanlert has pointed out in 1975 that “it is hoped that drug education will be a more effective step towards kratom use control” [1], foreseeing the failure of the legislative measures in South Asian countries.

Kratom pharmacology itself is complex and requires future research: this compound in fact acts on opioid as well as on dopaminergic, serotonergic, GABAergic, and adrenergic systems [17, 19]. Therefore, subjective effects are very peculiar ranging from psychostimulant to sedative-narcotic. Pharmacological mechanisms responsible for several of its alkaloids activity deserve yet to be clearly established in future studies. Altogether, available data on kratom suggest caution: this unregulated plant could exhibit a serious harmful potential, far beyond any “therapeutic” desired effect; in parallel, its anxiolytic, antidepressant, and analgesic properties need to be better explored by scientific research works, like, for example, in large blind randomized controlled clinical trials [28].

Potential users who tend to self-medicate and health professionals working with them should be clearly aware of the risks associated with kratom consumption [92]. As it emerged from this and other previous studies [93], kratom is advertised and sold online as “cheaper alternative” to traditional opioid replacement therapies, as a painkiller for chronic pain, or as an anxiolytic remedy in psychiatric population, with no need of medical prescription or supervision. This encourages a tendency to self-medicate and could become a serious problem for unaware doctors prescribing medication to a patient who is a regular kratom user or in case of acute intoxication related to the substance. Another issue of concern is the action of other isolated compounds (e.g., speciociliatine, speciogynine, and paynantheine) whose effects were not inhibited by naloxone in animal studies [22, 23], meaning a potentially very difficult management in case of overdose. Therefore, in this scenario, the risk of adverse reactions or possible misdiagnosis might be very high. It might also be worth adding that kratom use is not detected by conventional drug screening tests as advanced tests, like liquid chromatography-tandem or ion-mass spectrometry, are required [7]. We also reiterate here the importance of focusing and asking direct questions on the nature and patterns of drug intake, including medical products diversion and consumption of NPS, during clinical assessments [94]. Given that nowadays polydrug use represents the norm rather than the exception, especially in emergency settings [90], the simultaneous use of drugs should also be promptly identified, investigated, and well discriminated. Future studies should explicitly examine the effects of the combination in complex patterns of polydrug intake, including kratom, to fully understand the synergistic effects and associated clinical and toxicological implications. Attention should also be paid to the motivations behind such behaviours, social norms, stigma, availability, and other lifestyle factors.

Surely, a possible limitation of our analysis could be given by the fact that publicly available websites, fora, and similar sources were also considered and included together with a systematic literature review. One could wonder about the limitations of carrying out a risk assessment of a drug while taking into account also the online comments. It may be inappropriate to trust information obtained from the Internet without independent verification and we did not have any possibility here to ascertain if the substance the online alleged drug users were taking was indeed kratom. On the other hand, online reports about kratom seem genuine and many users illustrate their detailed experiences as proper experiments on themselves. Thus, in the lack of relevant peer-reviewed data, the online monitoring seems to be indeed a very useful method to obtain preliminary information about new and emergent phenomena [95]. Further, as demonstrated by the outcomes of this study, a better international collaboration is necessary to tackle this rapidly growing drug trend.

## Figures and Tables

**Box 1:**
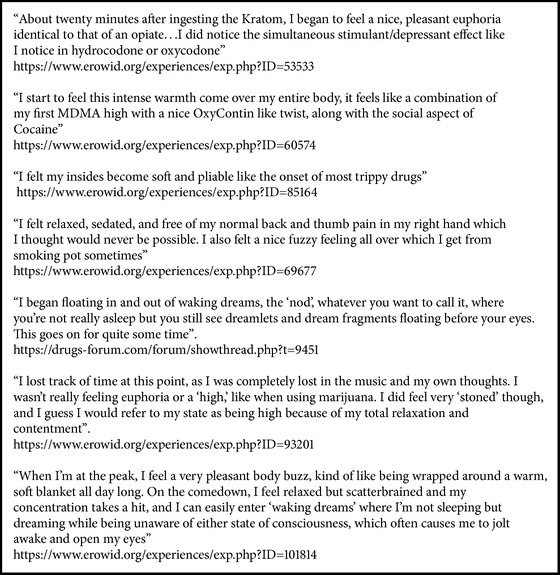
**Box 1: **Qualitative analysis of kratom users' experiences.

**Table 1:** Report of adverse/toxicological effects of kratom.

Short time use effects	Nausea, constipation, sleep problems, temporary erectile dysfunction, itching, or sweating

Long time use effects	Anorexia, dry mouth, problems in diuresis, darker skin, and hair loss

Withdrawal symptoms	Hostility, aggression, aching of muscles and bones, jerky movements of the limbs, anorexia and weight loss, and insomnia

Infrequent effects	Seizures (individuals using high doses of kratom, either alone or combined with other drugs), intrahepatic cholestasis, psychotic symptoms, Adult Respiratory Distress Syndrome, and hypothyroidism

Fatalities	Kratom mixed with other substances: O-desmethyltramadol; propylhexedrine; over-the-counter cold medications and benzodiazepines; venlafaxine, diphenhydramine, and mirtazapine; zopiclone, citalopram, and lamotrigine
